# 

**Published:** 1874-03

**Authors:** 


### Books and Pamphlets Received.

A Treatise on Therapeutics, comprising Materia Medica and Toxicology, with especial reference to the application of the Physiological action of Drugs to Clinical Medicine. By H. C. Wood, Jr., M. D. Philadelphia: J. B. Lippincott & Co., 1874 Buffalo: Martin Taylor.

Galvano-Therapeutics. A revised Reprint of A Report made to the Illinois State Medical Society. By David Prince, M. D. Philadelphia: Lindsay & Blakiston, 1873.

Annual Report of the Supervising Surgeon of the Marine Hospital Service of the U. S., for the fiscal year 1873. John M. Woodworth, M. D. Washington: Government Printing Office, 1873.

Lectures on the Clinical Uses of Electricity, delivered in University College Hospital. By J. Russell Reynolds, M. D., T. R. S. Philadelphia· Lindsay & Blakiston, 1874.

The Physicians Dose and Symptom Book. By Jno. H. Wythes, A. M. M. D· Eleventh Edition. Philadelphia: Lindsay & Blakiston, 1874.

Report of the Resident Physician of Brigham Hall, A Hospital for the Insane, for the year 1874.

Constitution and By-Laws of the Association of the Alumni of the Albany Medical College.

Medical and Pharmaceutical Notes. By Edward R. Squibb, M. D.

Contributions to the Study of Yellow Fever. By J. M. Toner, M. D., and John M. Woodworth, M. D.

THE BUFFALO

### Medical and Surgical Journal

PUBLISHED MONTHLY,

**Containing Original Articles, Reports of Medical Societies and Hospitals, Editorial, Reviews, Correspondence, Army News, etc., etc; including the usual variety of Medical Periodical Publications. Specimen copies sent on application.**

Address Buffalo Medical & Surgical Journal, Buffalo, N. Y.

TERMS OF SUBSCRIPTION, - - $3.00 PER YEAR, IN ADVANCE.

